# Persistent Bacterial and Fungal Community Shifts Exhibited in Selenium-Contaminated Reclaimed Mine Soils

**DOI:** 10.1128/AEM.01394-18

**Published:** 2018-08-01

**Authors:** Carla E. Rosenfeld, Bruce R. James, Cara M. Santelli

**Affiliations:** a Department of Earth Sciences, University of Minnesota, Twin Cities, Minneapolis, Minnesota, USA; b BioTechnology Institute, University of Minnesota, Twin Cities, St. Paul, Minnesota, USA; c Department of Environmental Science and Technology, University of Maryland, College Park, Maryland, USA; University of Tennessee and Oak Ridge National Laboratory

**Keywords:** metalloids, metals, microbial ecology, microbiome, pollution

## Abstract

Mining and other industrial activities worldwide have resulted in Se-enriched surface soils, which pose risks to human and environmental health. Although not well studied, microbial activity can alter Se bioavailability and distribution, even in oxic environments. We used high-throughput sequencing to profile bacterial and fungal communities inhabiting mine soils in southeastern Idaho, comparing mined and unmined locations within two reclaimed phosphate mine areas containing various Se concentrations. The goal was to determine whether microbial communities differed in (i) different mines, (ii) mined areas compared to unmined areas, and (iii) various soil Se concentrations. Though reclamation occurred 20 to 30 years ago, microbial community structures in mined soils were significantly altered compared to unmined soils, suggesting persistent mining-related impacts on soil processes. Additionally, operational taxonomic unit with a 97% sequence similarity cutoff (OTU_0.03_) richness and diversity were significantly diminished with increasing Se, though not with other geochemical parameters, suggesting that Se contamination shapes communities in favor of Se-tolerant microorganisms. Two bacterial phyla, Actinobacteria and Gemmatimonadetes, were enriched in high-Se soils, while for fungi, Ascomycota dominated all soils regardless of Se concentration. Combining diversity analyses and taxonomic patterns enables us to move toward connecting physiological function of microbial groups to Se biogeochemical cycling in oxic soil environments.

**IMPORTANCE** Selenium contamination in natural environments is of great concern globally, and microbial processes are known to mediate Se transformations. Such transformations alter Se mobility, bioavailability, and toxicity, which can amplify or mitigate Se pollution. To date, nearly all studies investigating Se-microbe interactions have used culture-based approaches with anaerobic bacteria despite growing knowledge that (i) aerobic Se transformations can occur, (ii) such transformations can be mediated by microorganisms other than bacteria, and (iii) microbial community dynamics, rather than individual organismal activities, are important for metal(loid) cycling in natural environments. We examined bacterial and fungal communities in Se-contaminated reclaimed mine soils and found significant declines in diversity at high Se concentrations. Additionally, we identified specific taxonomic groups that tolerate excess Se and may be useful for bioremediation purposes. These patterns were similar across mines of different ages, suggesting that microbial community impacts may persist long after physicochemical parameters indicate complete site recovery.

## Abstract (precis)

Selenium contamination in natural environments is of great concern globally, and microbial processes are known to mediate Se transformations. Such transformations alter Se mobility, bioavailability, and toxicity, which can amplify or mitigate Se pollution. To date, nearly all studies investigating Se-microbe interactions have used culture-based approaches with anaerobic bacteria despite growing knowledge that (i) aerobic Se transformations can occur, (ii) such transformations can be mediated by microorganisms other than bacteria, and (iii) microbial community dynamics, rather than individual organismal activities, are important for metal(loid) cycling in natural environments. We examined bacterial and fungal communities in Se-contaminated reclaimed mine soils and found significant declines in diversity at high Se concentrations. Additionally, we identified specific taxonomic groups that tolerate excess Se and may be useful for bioremediation purposes. These patterns were similar across mines of different ages, suggesting that microbial community impacts may persist long after physicochemical parameters indicate complete site recovery.

## INTRODUCTION

Though Se is an essential micronutrient for animals and bacteria, it can be a contaminant that poses significant ecological risk with lasting ecosystem-scale impacts in a wide variety of environments (1). Se is a naturally occurring trace element commonly associated with sulfur-containing minerals, and its distribution varies widely across terrestrial and marine ecosystems (2). Numerous human activities alter the natural Se cycle, resulting in Se contamination of natural environments. Such activities include mining, smelting, coal combustion, agricultural irrigation, and glass, electronics, and petroleum industries (3). In excess, Se can cause severe birth defects, reproductive failure, and local extinctions in aquatic wildlife (4). Additionally, it can cause neurological disturbances, bone deformations, and nervous system abnormalities in animals, including humans (5). In the United States, Se has recently gained recognition as an important environmental pollutant, including the development of an aquatic life criterion (6). No regulations or guidelines exist for Se contamination of U.S. soils, despite their ability to act as persistent Se sources in sensitive terrestrial and aquatic ecosystems (7).

In soils, Se can exist in many different forms, depending on its oxidation state and ligand environment, both of which dictate its potential movement throughout the environment and its bioavailability to plants (the primary Se source for land animals) and microorganisms. Se has four oxidation states, −II, 0, IV, and VI. In oxic environments (e.g., surface soils), Se(VI) and Se(IV) dominate, forming the water-soluble oxyanions selenate (SeO_4_^2−^) and selenite (SeO_3_^2−^). Conversely, the more reduced species [Se(−II) and Se(0)], which are expected to dominate in anoxic environments, tend to be insoluble or volatile [though some organic Se(−II) forms, such as selenocysteine, are soluble], therefore limiting food chain access and potential toxicity in Se-contaminated ecosystems (8).

Various abiotic and biotic processes can mediate Se oxidation-reduction (redox) transformations in soils, which in turn influence the form and subsequent bioavailability of Se in an ecosystem. Abiotic soil factors influencing Se speciation in soils include O_2_ content, pH, and the presence and types of mineral surfaces on which it may be adsorbed or react (9, 10). Microbial processes also influence Se speciation and subsequent mobility and bioavailability in soils. Of particular interest are microorganisms that can remove excess Se from the food chain and water supplies via methylation (and subsequent volatilization) or reduction to elemental Se(0) (11). Anaerobic microbial transformations of Se have been relatively well studied, with many known bacteria able to reduce Se via assimilatory reduction [i.e., reducing Se(IV) or Se(VI) to organic Se(−II) forms via Se-amino acid production] or dissimilatory reduction [i.e., anaerobic respiration using Se(IV) or Se(VI) as a terminal electron acceptor] (12). In addition to anaerobic processes, microbially mediated aerobic Se(IV,VI) reduction can occur, either directly to detoxify it or indirectly via reaction with other reductants (13, 14), though the mechanisms driving these processes, as well as the organisms capable of mediating them, have been the focus of substantially less research.

Considering the importance of microorganisms in the Se biogeochemical cycle and the implications of altering Se speciation on human and environmental health, there is a clear knowledge gap surrounding microbial-Se interactions. While culture-based approaches have succeeded at identifying individual Se-tolerant and/or Se-transforming organisms (15–19), information regarding whole-microbial-community responses to Se contamination is lacking. Most studies show that microbial abundance, activity, diversity, and distribution are severely depressed in metal(loid)-contaminated soils compared with uncontaminated soils (20, 21). Similarly, reclaimed soils in mine sites have been shown to have lower microbial diversity and metabolic activity than in unmined sites, though these results are highly dependent on a number of factors, including site location, mining and reclamation methods, dominant vegetation, and number of years after reclamation (22, 23). Some studies have even observed shifts in microbial population structures from bacterium- to fungus-dominated biomass resulting from metal contamination (21, 24, 25), though others have observed the opposite change (26, 27). Accurately measuring bacterium-to-fungus ratios in soils is notoriously difficult (28). Despite their overall reduction in microbial activity, biomass, and diversity, metal- and mining-impacted environments still contain diverse communities of metal-tolerant bacteria, archaea, and microeukaryotes (e.g., fungi and algae) (29). Microbial community interactions and dynamics could thus be important in regulating Se chemical speciation and bioavailability in soils.

Phosphorus mining activities in the “Phosphate Patch” of southeast Idaho, USA, an area that overlies the Phosphoria Formation, have resulted in the release of several elements of concern (30). Of primary concern is Se, which has been linked to a series of livestock deaths in the area, as well as adverse effects on aquatic invertebrates and fish in the Blackfoot River and its tributaries (31). Elevated concentrations of Se have also been measured in soils, surface waters, and vegetation (32), though how the soil microbial community is interacting with, or is influenced by, this contamination is unclear. Microbial community dynamics may mediate metalloid speciation in soils, but to date, the only studies investigating microbial communities in Se-impacted natural environments have focused on communities in anaerobic environments (15, 33–35). The resident communities in Se-contaminated oxic soil environments remain completely unknown. Furthermore, no known studies have examined the fungal community in Se-impacted environments, despite the existing knowledge of fungal activity influencing Se biogeochemistry (17, 36). The objective of the current study was to comprehensively investigate the bacterial and fungal communities in Se-impacted surface soils in two reclaimed phosphorus mines of southeastern Idaho. We aimed to determine whether (i) different mine areas host unique microbial communities or if similar communities are present in both mine areas, (ii) mined and unmined soils host different microbial communities, and (iii) microbial communities in soils with elevated Se are distinct from communities in soils with low Se. We measured diversity indices, community structure parameters, and taxonomy profiles, in combination with bulk soil geochemistry over 2 years. Based on these data, we developed an understanding of the overall microbial community in these reclaimed mine soils and its relationship to mining history and Se concentration in the soils.

## RESULTS

### Soil geochemistry.

Several locations in both the Champ Mine (CM; 6 previously mined and reclaimed locations plus one nonmined reference location) and Mountain Fuel Mine (MFM; 7 reclaimed locations plus one nonmined location) were sampled over two summers (see Fig. S1 in the supplemental material) in an attempt to encompass the range of biological, geochemical, and physical conditions on the two mine sites. A particular emphasis was made to sample soils with varied Se concentrations, as Se enrichment is a concern in the area (30). Soil Se concentrations ranged from 1 to 2 mg · kg^−1^ soil in the nonmined reference sites and from 3 to 77 mg · kg^−1^ in the mined/reclaimed sites (Table S1 and Fig. S2). While the current study did not investigate Se speciation in the soils, previous work by Ryser et al. (37) determined that soils developing from Se-bearing shale in this region were dominated by a mixture of Se(IV) and Se(VI) formed through oxidative weathering of the more reduced Se(−II) and Se(0) minerals contained within the shale. The concentrations in the current study were determined using hydrofluoric (HF) acid digestions, which measures total metal(loid) concentrations in soils. This measure is likely an overestimate of the bioavailable fraction of Se in these soils, which may be more accurately assessed in the future with hot-water, phosphate, or other organic-ligand extractions. Se concentrations in the soils were significantly positively correlated (*P* < 0.05) with soil concentrations of As (*R*^2^_adjusted_ = 0.55), Cd (*R*^2^_adjusted_ = 0.74), Ni (*R*^2^_adjusted_ = 0.70), and Zn (*R*^2^_adjusted_ = 0.67) (Fig. S2). In contrast, negative correlations between Se and total soil Fe (*R*^2^_adjusted_ = 0.01) and Mn (*R*^2^_adjusted_ = 0.41) were observed. Although the Se correlations with Fe and Mn were statistically significant (*P* < 0.05), these relationships showed substantially more variation than for the other metals, as indicated by the decreased correlation coefficients (*R*^2^_adjusted_). Soil pH ranged from 6.4 (CM-R) to 8.1 (CM-BP1), and year-to-year variability was minimal, with most soils remaining within 0.5 pH units from 2015 to 2016 (Table S1). Organic C ranged from 7.5 g · kg^−1^ (MFM-VD2, 2016) to 73.2 g · kg^−1^ (CM-BP1, 2016), and total N ranged from 0.5 g · kg^−1^ (MFM-NPD2, 2015) to 7.3 g · kg^−1^ (CM-BP1, 2016, and CM-ED2, 2015). Increasing total N was significantly correlated with increasing Se (*R*^2^_adjusted_ = 0.3, *P* = 5 × 10^−7^), though the correlations of Se with pH (*R*^2^_adjusted_ = 0.04) and organic C (*R*^2^_adjusted_ = 0.08) were very poor (Fig. S2).

Soils with average Se concentrations greater than 30 mg · kg^−1^ were categorized as “high Se,” while locations with average Se concentrations less than 30 mg · kg^−1^ were categorized as “low Se.” This concentration value was selected as the cutoff between high and low Se based on typical concentrations in Se-contaminated environments (3) and laboratory studies showing significant fungal growth inhibition and aerobic Se reduction of both Se(IV) and Se(VI) (17). Across the 2 years, 8 out of 26 mined soil samples were categorized in the high-Se category. Most samples categorized as low Se in 2015 (i.e., <30 mg · kg^−1^) remained categorized as low Se in 2016, though there were two exceptions in each mine (Table S1). In the Champ Mine, soils collected from sample location CM-ED5 had an average of ∼9 mg · kg^−1^ in 2015 and ∼77 mg · kg^−1^ in 2016, and soils collected from sample location CM-BP1 increased from ∼16 mg · kg^−1^ in 2015 to ∼61 mg · kg^−1^ in 2016. In the Mountain Fuel Mine, soils collected from sample location MFM-NPD3 had an average of ∼5 mg · kg^−1^ in 2015 and ∼39 mg · kg^−1^ in 2016, whereas soils collected from sample location MFM-SD1 averaged ∼39 mg · kg^−1^ in 2015 but only ∼19 mg · kg^−1^ in 2016. This year-to-year variability is consistent with previous documentation of highly heterogeneous Se distribution in reclaimed Phosphoria Formation mine soils resulting from nonuniform distribution and incorporation of Se-bearing waste shale into the reclaimed soils (38, 39).

### DNA sequence characterization.

In total, 7.9 × 10^6^ sequences of the bacterial and archaeal 16S rRNA gene V4 region and 7.6 × 10^6^ sequences of the fungal internal transcribed spacer 1 (ITS1) region were retained after quality control and negative-control removal. This represented 52% of the initial raw V4 sequence reads and 70% of the initial raw ITS1 sequence reads from our MiSeq library. Following *de novo* clustering into operational taxonomic units (OTUs; 97% sequence similarity for 16S and ITS), these reads constituted 6.0 × 10^4^ bacterial OTU, 950 archaeal OTU, and 7.3 × 10^3^ fungal OTU. Because the archaeal OTUs represent a vast minority of the V4 sequences in our data set (average relative abundance of archaeal sequences, ∼3%), we limited further analysis to bacterial sequences only (Fig. S3).

### Influence of mine history and soil geochemistry on microbial α-diversity.

**FIG 1:**
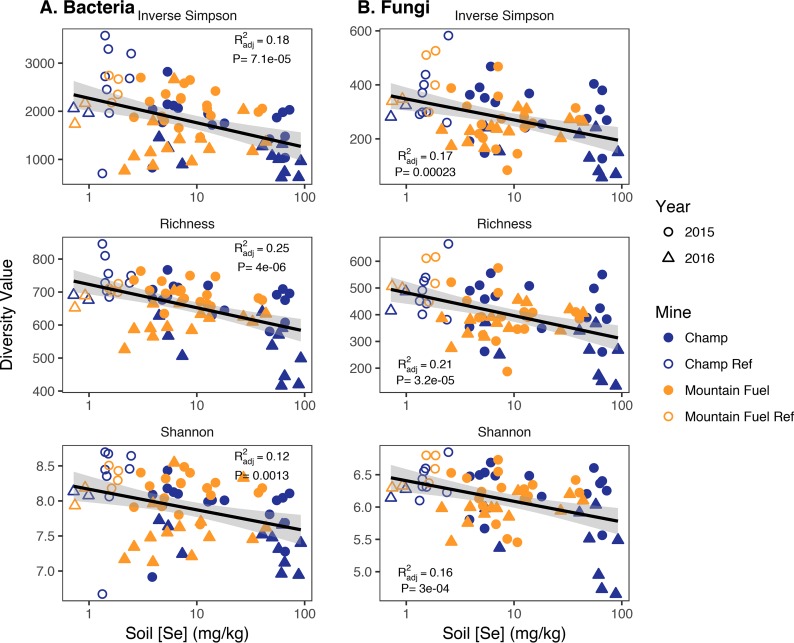
Linear regressions (black lines) of three diversity measures (inverse Simpson diversity index, OTU richness, and Shannon diversity index), as a function of soil [Se] for bacteria (A) and fungi (B) in the Champ Mine (dark blue, closed symbols), Champ references (dark blue, open symbols), Mountain Fuel Mine (yellow, closed symbols), and Mountain Fuel reference (yellow, open symbols). Each data point represents an individual sample taken in either 2015 (circles) or 2016 (triangles). Gray shading is the 95% confidence interval calculated for the linear regressions. Pearson correlation coefficients (*R*^2^_adj_) and *P* values are listed in the top right or bottom left of each panel. Regressions were considered statistically significant with *P* values of <0.05.

**FIG 2:**
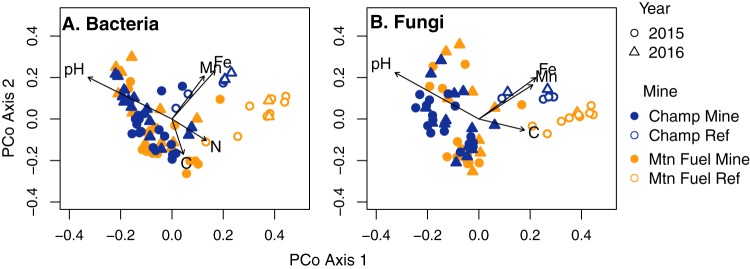
Principal-coordinate analysis of bacterial (A) and fungal (B) β-diversity in Champ Mine, Mountain (Mtn) Fuel Mine, Champ reference, and Mountain Fuel reference sites. Axis 1 represents 17% and 11% of the ordination variation for bacteria and fungi, respectively. Axis 2 explains 10% and 6% of the variation for bacteria and fungi, respectively. Operational taxonomic unit (OTU) abundances were Hellinger transformed prior to dissimilarity calculation. Black lines indicate environmental factors with significant correlations (*P*_adj_ < 0.05) to the bacterial and fungal OTU ordinations. Lines are scaled by correlation coefficient of the environmental variable (Table S3).

α-Diversity, as assessed by the inverse Simpson diversity index, OTU richness, and the Shannon diversity index (H′), all indicated similar trends, regardless of the metric used (Fig. 1). All three indices showed that α-diversity was greater for both bacteria and fungi in the reference (nonmined) areas than in the mined areas and that it decreased significantly with increasing Se concentration. For all three diversity indices, Se concentration in the soil explained ∼12 to 25% of the variability (*R*^2^_adjusted_ = 0.12 to 0.25; *P* < 0.002) in bacterial diversity and 16 to 21% of the variability (*R*^2^_adjusted_ = 0.16 to 0.21; *P* < 0.001) in fungal diversity (Fig. 2). Most other geochemical factors did not have a statistically significant correlation with the three α-diversity metrics (*P* > 0.05; organic C, Fe) and/or the correlation was very poor (*R*^2^_adjusted_ < 0.1; pH, total N) for both bacteria and fungi (Table S2). The primary exception to this was Mn, which had a statistically significant positive correlation with the three metrics for both bacteria and fungi, though this was likely a result of very high Mn concentrations in the reference samples compared to mined samples, further indicating that diversity was greater in unmined soils than in mined soils.

### Influence of location, mine history, and geochemical factors on microbial community structure (β-diversity).

Principal-coordinate analysis (PCoA) was performed on both bacterial and fungal OTU data sets to cluster samples based on the similarity of their microbial community structures. PCoA based on Bray-Curtis distances of Hellinger-transformed OTU relative abundances showed that both bacteria and fungi largely clustered into two groups based on whether or not the sampling location had previously been mined (bacteria, *P* = 0.001, *R*^2^ = 0.12; fungi, *P* = 0.001, *R*^2^ = 0.1; Table 1). The Bray-Curtis distance gives a measure of community composition dissimilarity based on relative OTU abundance, and the Hellinger transformation gives low weights to rare OTUs. Using a permutational multivariate analysis of variance (PERMANOVA), we tested the statistical significance of differences in bacterial and fungal community compositions in relation to mining history, sample location, year collected, and Se concentration in the soils. Though PERMANOVA (Table 1) indicated that the mine area (Champ or Mountain Fuel) was statistically significant (*P* = 0.001), the percent variation of community β-diversity explained by mine area, as described by the PERMANOVA *R*^2^ value, was very low (bacteria, *R*^2^ = 0.04; fungi, *R*^2^ = 0.05), suggesting that the community β-diversity in the two mine areas was relatively similar (Fig. 2). Similarly, in our comparison of fungal and bacterial β-diversity in sampling locations with high Se (>30 mg/kg) and low Se (<30 mg/kg), or in samples collected during different years (2015 or 2016), we also observed statistically significant PERMANOVA results, though the *R*^2^ values indicated that these factors explained minimal variation in the community β-diversity (Table 1).

**TABLE 1:** PERMANOVA and PERMDISP results based on Bray-Curtis dissimilarities of Hellinger-transformed OTU abundance data of bacteria and fungi

Comparison group	PERMANOVA			PERMDISP^*a*^			
	*R*^2^	Pseudo-*F*	*P*^*b*^	Distance factor 1	Distance factor 2	*F* value	*P*^*b*^
Bacteria							
Mining history (mined or reference)	0.12	10.41	0.001	0.4	0.38	0.78	0.4
Mine area (Champ or Mountain Fuel)	0.043	3.27	0.001	0.45	0.39	22.5	0.001
Yr (2015 or 2016)	0.048	3.65	0.001	0.41	0.42	0.02	0.9
Se status (low or high)	0.045	3.44	0.001	0.42	0.4	0.6	0.4
Fungi							
Mining history (mined or reference)	0.1	7.24	0.001	0.54	0.49	14.2	0.003
Mine area (Champ or Mountain Fuel)	0.045	3.27	0.001	0.56	0.53	4.6	0.04
Yr (2015 or 2016)	0.039	2.83	0.001	0.54	0.55	0.6	0.4
Se status (low or high)	0.037	2.65	0.001	0.55	0.53	3.5	0.06

a PERMDISP distance is the average distance of individual β-diversity observations to the group median (centroid). Factor 1 refers to the first factor listed in parentheses for each comparison listed on left. Factor 2 refers to the second factor listed in parentheses for each comparison listed on left.

b *P* values of <0.05 are considered statistically significant.

In addition to the PERMANOVA, we measured the β-dispersion using PERMDISP to determine the average distance of individual observations to the group centroid (median). This analysis relies on an *F*-statistic to determine whether observed shifts are driven by multivariate heterogeneity, with greater *F* values indicating statistically significant β-dispersion for different groups. Fungal β-dispersion (distance to group centroid) was significantly less in reference sites than in mined sites (*F* = 14.2, *P* = 0.003; Table 1), though there was no significant difference in distance to the group centroid for the bacterial communities in mined and reference sites (*F* = 0.78, *P* = 0.4; Table 1). Additionally, both bacteria and fungi had significantly shorter distances to the group centroid in Mountain Fuel (mine and reference) sites than in Champ (mine and reference) sites (bacteria, *F* = 22.5, *P* = 0.001; fungi, *F* = 4.6, *P* = 0.04; Table 1). Neither bacterial nor fungal community β-dispersion was significantly different for samples collected in different years (bacteria, *F* = 0.02, *P* = 0.9; fungi, *F* = 0.6, *P* = 0.4; Table 1). With respect to Se status, the bacterial community β-dispersion was not significantly different (*F* = 0.6, *P* = 0.4; Table 1), though the fungal community β-dispersion trended toward shorter distance to the group centroid in high-Se soils (*F* = 3.5, *P* = 0.06; Table 1).

In comparing the geochemical factors to bacterial and fungal community structures using PCoA, we included pH and organic C, Fe, Mn, N, and Se concentrations. The other metal(loids) (As, Cd, Ni, and Zn) were excluded from this analysis because they were all significantly and positively correlated with Se concentration in the soils (Fig. S2). Both bacterial and fungal ordinations were significantly (*P* < 0.05) correlated with several geochemical factors, with pH the highest ranking factor for both (bacteria, *R*^2^ = 0.52, *P* = 0.001; fungi, *R*^2^ = 0.48, *P* = 0.001; Fig. 2 and Table S3). Bacterial and fungal community structures were also significantly correlated with Fe (bacteria, *R*^2^ = 0.29, *P* = 0.001; fungi, *R*^2^ = 0.27, *P* = 0.001) and Mn (bacteria, *R*^2^ = 0.21, *P* = 0.003; fungi, *R*^2^ = 0.22, *P* = 0.001; Fig. 2 and Table S3). Organic C was also significantly correlated with bacterial and fungal community structures, though the organic C correlation was substantially weaker than for Fe and Mn (bacteria, *R*^2^ = 0.11, *P* = 0.03; fungi, *R*^2^ = 0.11, *P* = 0.03; Fig. 2 and Table S3). Similarly, the bacterial community structure was weakly correlated with total N (*R*^2^ = 0.10, *P* = 0.03; Fig. 2 and Table S3). Neither bacterial nor fungal community structures were significantly correlated with Se concentration (bacteria, *R*^2^ = 0.06, *P* = 0.13; fungi *R*^2^ = 0.07, *P* = 0.11; Table S3).

### Taxonomic profile of bacterial and fungal communities.

**FIG 3:**
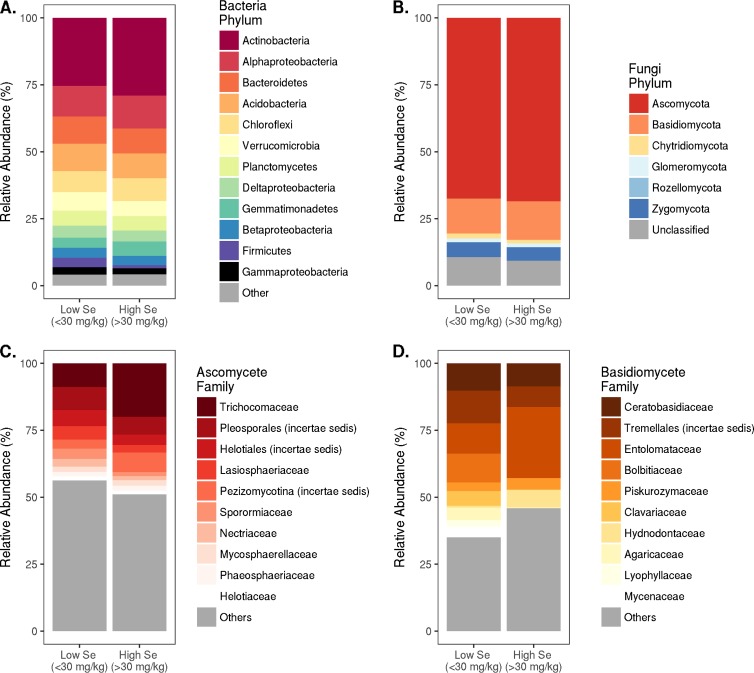
Taxonomic affiliation of bacterial (phylum level) (A) and fungal sequences at the phylum level (B) and family level (C and D) in high-Se (>30 mg/kg) and low-Se (<30 mg/kg) soils. A comparison of the bacterial and fungal taxonomic affiliations with respect to mined and reference soils and samples collected in 2015 and 2016 is presented in Fig. S4.

In addition to investigating community structure and diversity, we aimed to assess the taxonomic makeup of the bacterial and fungal communities in relation to the mining and Se stressors present in the Champ and Mountain Fuel sites. In total, 46 bacterial phyla were classified, with 1% of the bacterial sequences remaining unclassified (80% confidence cutoff) at the phylum level. With regard to high- and low-Se soils, Actinobacteria and Gemmatimonadetes had significantly increased relative abundances in high-Se soils, while no bacterial phyla had significantly increased relative abundances in low-Se soils (Table S4), though most bacterial phyla showed fairly similar relative abundances in both high and low-Se soils (Fig. 3A; see Table S4 in the supplemental material). Within the two phyla that were significantly enriched in the high-Se soils, Actinobacteria phylotypes were highly diverse, distributed across 24 orders, with sequences belonging to the Solirubrobacterales, Micrococcales, and Frankiales orders present in the greatest relative abundances of both high- and low-Se mined soils (Fig. S5). Conversely, Gemmatimonadetes phylotypes were highly constrained to a single order, Gemmatimonadales, with a small percentage classified as Longimicrobiales (Fig. S5).

Of the 12 bacterial phyla with greater than 1% relative abundance, several had significantly different relative abundances (Table S5) in mined (both high- and low-Se-containing) from those in unmined (only low-Se concentrations) soils (Alphaproteobacteria, Bacteriodetes, Acidobacteria, Verrucomicrobia, Planctomycetes, Betaproteobacteria, and Gammaproteobacteria) or by year sampled (Actinobacteria, Bacteriodetes, Verrucomicrobia, Planctomycetes, Deltaproteobacteria, Firmicutes, and Gammaproteobacteria). A statistically significant interaction effect of mining history and year was only observed for a single phylum, Acidobacteria (adjusted *P* = 0.002, Table S5).

**FIG 4:**
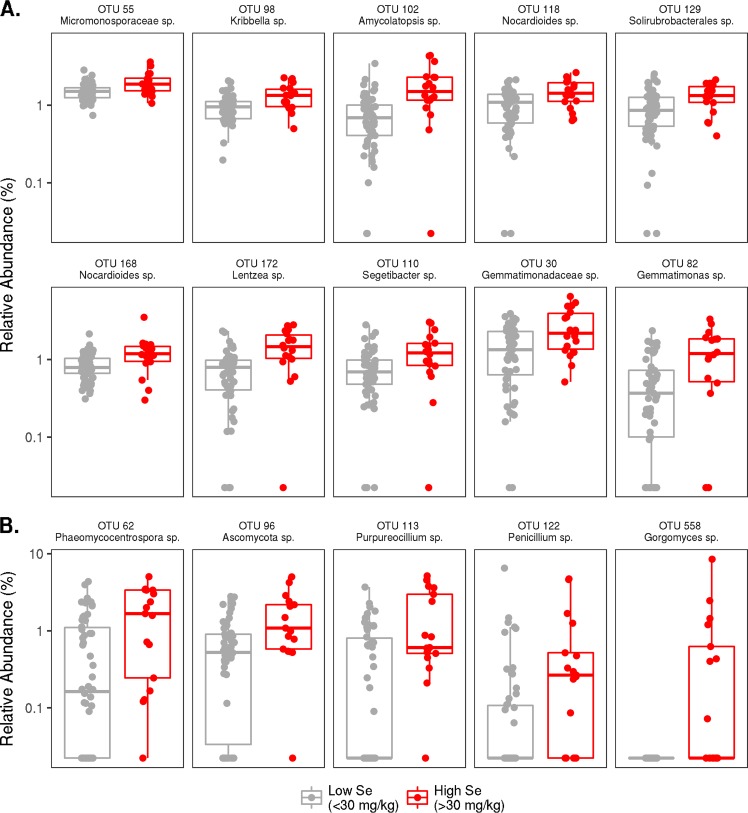
Bacterial (A) and fungal (B) operational taxonomic units (OTUs) present in significantly greater relative abundances (adjusted *P* < 0.05) in high-Se soils than in low-Se soils.

At the OTU_0.03_ level, 10 bacterial OTUs were present in significantly greater relative abundances (adjusted *P* < 0.05) in high-Se soils than in low-Se soils (Fig. 4A). For this analysis, only OTUs with a mean relative abundance greater than 0.1% were considered. The mean relative abundances of these OTUs were in the range of 0.1 to 1% in high-Se soils and 0.06 to 0.3% in low-Se soils (Table S6). Phylum-level classification showed that seven of these OTUs (OTUs 55, 98, 102, 118, 129, 168, and 172) were Actinobacteria, two OTUs (OTUs 30 and 82) were Gemmatimonadetes, and one (OTU 110) was a Bacteroidetes. The most abundant of these OTUs (OTU 30), with a mean relative abundance in high-Se soils of 1% compared to 0.03% in low-Se soils, was classified at the family level as Gemmatimonadaceae, but it could not be classified beyond that taxonomic level (Table S6). Other OTUs present in significantly greater relative abundances in high-Se soils were all classified at the genus level except OTU 129 (family FFCH13075 in the Solirubrobacteriales order, Actinobacteria phylum), and OTU 55 (Micromonosporaceae family in the Micromonosporales order, Actinobacteria phylum). The genera that were significantly enriched in high-Se soils from the Actinobacteria phylum were Kribbella (OTU 98), Amycolatopsis (OTU 102), Nocardioides (OTUs 118 and 168), and Lentzea (OTU 172), while Segetibacter (OTU 110) is a member of the Bacteroidetes phylum, and Gemmatimonas (OTU 82) is a member of the Gemmatimonadetes phylum (Table S6).

For fungi, the vast majority of total DNA sequences (regardless of sample type) were classified as belonging to the Ascomycota phylum, followed by Basidiomycota. All other fungal phyla (Chytridiomycota, Glomeromycota, Rozellomycota, and Zygomycota) contributed minimally (2.3, 1.3, 0.02, and 5.0%, respectively), and 9.7% of the sequences remained unclassified (80% confidence cutoff) at the phylum level (Fig. 3B and Table S7). The relative abundances of fungal phyla were not significantly different in a comparison of high- or low-Se soils (Fig. 3B and Table S7) or by year sampled (Fig. S4B and Table S8). Only Zygomycota, of which 96% of the sequences belonged to the Mortierellaceae family, had a significantly different relative abundance in soils with different mining history, where the relative abundance in mined soils (3.8%) was substantially lower than that in reference soils (9.8%; *P* = 0.002; Table S8).

Within the Ascomycota phylum, the 10 most dominant families (Trichocomaceae, Pleosporales [incertae sedis], Helotiales [incertae sedis], Lasiosphaeriaceae, Pezizomycotina [incertae sedis], Sporormiaceae, Nectriaceae, Mycosphaerellaceae, Phaeosphaeriaceae, and Helotiaceae) comprised ∼50% of all Ascomycota sequences (Fig. 3C and S4C). None of these families had significantly different relative abundances in samples with different mining histories or by sampling years (Table S9). However, two of these dominant Ascomycota families were present with significantly different relative abundances in high- and low-Se mined soils (Table 2). The relative abundances of both Trichocomaceae and Pezizomycotina (incertae sedis) were significantly greater (adjusted *P* = 0.009 for both families) in high-Se soils (16% and 6%, respectively) than in low-Se soils (7.9% and 2%, respectively).

**TABLE 2:** Relative abundances and associated adjusted *P* value from one-way analysis of variance for top fungal families of Ascomycota and Basidiomycota in low- and high-Se soils

Family by phylum	Relative abundance (%)		Adjusted *P* value^*a*^
	Low-Se soils	High-Se soils	
Ascomycota			
Pleosporales (incertae sedis)	10.4	8.9	0.59
Trichocomaceae	8.7	16.0	0.005
Lasiosphaeriaceae	5.3	3.1	0.15
Helotiales (incertae sedis)	5.9	3.9	0.25
Pezizomycotina (incertae sedis)	3.0	6.4	0.003
Sporormiaceae	3.8	1.3	0.06
Mycosphaerellaceae	2.3	2.8	0.93
Nectriaceae	2.8	1.6	0.06
Helotiaceae	1.8	1.9	0.82
Phaeosphaeriaceae	1.6	2.8	0.65
Basidiomycota			
Ceratobasidiaceae	12.9	13.0	0.93
Entolomataceae	7.6	12.0	0.35
Tremellales (incertae sedis)	13.4	8.2	0.51
Hydnodontaceae	0.8	9.9	0.28
Bolbitiaceae	5.9	0.01	0.51
Piskurozymaceae	4.3	3.3	0.51
Clavariaceae	5.6	0.001	0.21
Lyophyllaceae	3.5	0.001	0.54
Mycenaceae	3.4	0.0004	0.35
Agaricaceae	4.2	0.50	0.25

a Adjusted *P* values of <0.05 are considered statistically significant.

Within the Basidiomycota, the 10 most abundant families (∼60% of the Basidiomycota sequences) were dominated by just three families (Ceratobasidiaceae, Entolomataceae, and Tremellales [incertae sedis]; Fig. 3D and S4D). The remaining seven families (Hydnodontaceae, Bolbitiaceae, Piskurozymaceae, Clavariaceae, Lyophyllaceae, Mycenaceae, and Agaricaceae) encompassed 2 to 4.5% of the total Basidiomycota sequences. While there were no statistically significant differences in relative abundances of basidiomycete families in high- or low-Se soils, five families (Bolbitiaceae, Clavariaceae, Lyophyllaceae, Mycenaceae, and Agaricaceae) were reduced to <1% relative abundance in high-Se mined soils from 3 to 7% in low-Se mined soils (Table 2). As with the dominant ascomycete families, relative abundances of the dominant basidiomycete families were quite consistent among mined and reference soils and sampling years (Table S10), and only Tremellales (incertae sedis) and Mycenaceae had significantly different relative abundances in mined soils (9% and 0.7%, respectively) from those in reference soils (20% and 8%, respectively; adjusted *P* = 0.002).

At the OTU_0.03_ level, five fungal OTUs (OTUs 62, 96, 113, 152, 558) were present in significantly greater relative abundances (adjusted *P* < 0.05) in high-Se than in low-Se soils (Fig. 4B and Table S11), and all were classified as Ascomycota. For this analysis, only OTUs with a mean relative abundance greater than 0.1% were considered. The mean relative abundances of these OTUs ranged from 0.2 to 0.6% in high-Se soils and 0 to 0.1% in low-Se soils (Table S11). Four of the OTUs were assigned consensus taxonomy at the genus level (Phaeomycocentrospora sp. [OTU 62], Purpureocillium sp. [OTU 113], Gorgomyces sp. [OTU 558], and Penicillium sp. [OTU 122]), and one remained unclassified beyond the phylum level (unclassified Ascomycota [OTU 96]).

## DISCUSSION

### Influence of mine history and soil geochemistry on microbial α- and β-diversity.

Microbial communities are known to be sensitive to changes in their local environmental conditions, particularly in mining-impacted areas (40, 41). Mining of the areas included in this study occurred during 1982 to 1985 (Champ Mine) and 1985 to 1993 (Mountain Fuel Mine), and reclamation for the two mines was completed in 1986 (Champ Mine) and 1995 (Mountain Fuel Mine) (30). The mining and reclamation processes for both mines were similar (30), and despite mining and reclamation occurring at different times, there was no clear difference between bacterial or fungal community structures (i.e., β-diversity) measured in soils collected from the two mine areas. Conversely, both bacteria and fungi had significantly different β-diversity in locations that were mined than in locations that were not mined. One reason for this may be the differences in vegetative communities growing on the mined and unmined soils (42). Lewis et al. (43) observed similar impacts on the bacterial communities in mined bauxite soils of Jamaica, with significantly lower bacterial diversity (α and β) and altered taxonomy even after more than 20 years of reclamation. Similarly, a study investigating bacterial communities in reclaimed coal mine soils also observed significant losses in bacterial community diversity compared to undisturbed soils, despite stabilization of soil geochemical factors within the same time frame (44). A third study, however, found a similar initial decrease in bacterial biodiversity after coal mine reclamation, though after 15 to 20 years, the bacterial diversity and taxonomic structure had returned to premining levels (45).

While the effects of mining on fungal communities are not as well studied, one study using lipid biomarker analysis observed decreased fungal biomass even 20 years following surface mine reclamation (23). However, just as the bacterial studies show inconsistent trends in longer-term community effects, another study using lipid biomarkers observed fungal recovery to predisturbance levels by 5 to 14 years postmining (22). Interestingly, a study of reforested mine soils showed these soils actually had greater fungal diversity than unmined reference soils 30 years after reclamation, despite initial declines in diversity (46). Both bacterial and fungal diversity and succession postdisturbance have been shown to be highly dependent on vegetation type and/or soil properties, which can vary depending on the length of time postreclamation, management strategies, and other environmental conditions (42). The variability in short- and long-term effects on microbial communities emphasizes the complexity of the relationship and impacts of these communities on environmental health and recovery, and it further suggests that microbial communities are highly site dependent and will be difficult to broadly compare across diverse sites. Future work may employ mesocosm or greenhouse-scale studies in order to investigate the mechanistic controls on microbial community composition in these soils. Regardless, the current study identified sustained decreases in the microbial diversity up to 30 years postreclamation, suggesting that these soils may have experienced a persistent, and perhaps irreversible, shift in ecosystem structure as a result of mining in this area.

This study did show that bacterial and fungal β-diversity were significantly correlated with pH in the soils, though it was not a significant factor for predicting the α-diversity of either group. Studies have previously found pH to be a significant factor for bacterial and/or fungal community structure (47, 48), though the pH range investigated is typically much wider (pH range in the current study, 6.4 to 8.1). Nutrient and contaminant bioavailability is highly related to soil pH, and in combination with optimal pH growth conditions, may contribute to the pH dependence of the microbial community structure. Conversely, the correlation with pH may be related to mining history, as the pH in mined soils was generally slightly greater than the pH of the unmined soils. Similar pH patterns have been observed previously in reclaimed surface mines (22) and may be related to the dilution of acidic topsoils with subsoils buffered at a higher pH.

Although significant changes in bacterial and fungal communities were observed in mined soils compared to unmined soils, β-diversity was, surprisingly, not significantly correlated with Se concentration in the mined soils. While overall community structure was stable, α-diversity for both bacteria and fungi was decreased in soils with elevated Se compared to soils with low Se. Since Se concentrations were positively correlated with As, Cd, Ni, and Zn concentrations in these soils, it is difficult to assess whether the driver of the α-diversity decline was due to Se, other metal(loid)s, or a combined effect of Se and other metal(loid)s. In the current study, Se concentrations in high-Se soils were substantially enriched compared to average noncontaminated soils in the United States (49). Relative to the concentrations in noncontaminated soils, the concentrations of other metal(loid)s (As, Cd, Ni, and Zn) were elevated, but they were not nearly as enriched as Se (49). Additionally, specific Se toxicity events have occurred in the region, suggesting that Se is mobile in these soil systems and the primary ecological concern rather than the other metal(loid)s (32). While the current analysis cannot rule out combined metal(loid) effects, Se contamination remains a significant risk in the region, and further work is necessary to establish a mechanistic understanding of the impacts of Se on soil microbial communities. Metal(loid) contamination is often associated with decreasing microbial taxonomic diversity compared to noncontaminated areas (50, 51), which has also been linked to a loss of key functions performed by specialized groups, such as arbuscular mycorrhizal fungi (52). However, these patterns are not always apparent, perhaps because of community adaptation to long-term contamination (53). Though the soils in the current study have been reclaimed for >20 years, it appears that the microbial communities have not adapted or fully recovered to premining composition. However, it is difficult to predict how these 2 years fit into the overall diversity patterns of the soils in the Champ Mine and Mountain Fuel Mine without longer-term sampling. Future work would be well suited to focus on the successional patterns of the microbial communities living in these reclaimed soils over a longer time period to fully understand the impacts of mine reclamation on taxonomic, and potentially, functional diversity.

### Taxonomic profiles of bacterial and fungal communities.

In addition to using OTU-based analyses, developing an understanding of the taxonomic profiles with respect to mining history and Se contamination can help us (i) place the current state of mine reclamation at the Champ and Mountain Fuel Mines in context of other mine reclamation projects, and (ii) develop hypotheses regarding the relationship between the physiological function of microbial groups to Se biogeochemical cycling in the soil environment. For example, Macur et al. (54) isolated multiple aerobic As(V)-reducing bacteria simultaneously with As(III)-oxidizing bacteria from unsaturated soil columns, suggesting that the taxonomic makeup was an important driver of functional capacity and can influence the metalloid speciation and mobility in this soil system. There were two bacterial phyla (Actinobacteria and Gemmatimonadetes) that displayed statistically significant increases in relative abundances in high-Se soils compared to low-Se soils. Actinobacteria have been shown previously to be tolerant of metal contamination (55). Additionally, both Actinobacteria and Gemmatimonadetes abundances have been positively correlated with As and Sb concentrations in contaminated soils (56). Of particular interest was a study by Gremion et al. (57) which showed that Actinobacteria phylotypes dominated RNA sequences collected from metal-contaminated bulk and rhizosphere soils. This suggests that Actinobacteria have a high tolerance of metal pollution in soils and may play a role in pollutant transfer from soils to plants. Several Actinobacteria strains are known to tolerate and/or transform high concentrations of Se, including those of Streptomyces sp. (58), Salana multivorans (59), and Microbacterium saperdae (60), as well as numerous strains in the Frankia genus (61). Though the current study did not investigate metabolic activity, nearly all of the bacterial OTUs significantly enriched in the high-Se soils were members of the Actinobacteria, suggesting that they may also play an important role in metabolic activity in the most contaminated areas of the reclaimed mines.

Along with Actinobacteria, Proteobacteria dominated all soils regardless of Se content, with the majority of Proteobacteria belonging to the Alphaproteobacteria class. Alphaproteobacteria are well-known members of soil microbial communities in prairie soils and have specifically been shown to dominate the Proteobacteria community in grasslands overlying previously mined soils (43) and in a stable selenate-reducing biofilm reactor (62). Currently, the vast majority of known Se-tolerant and/or Se-transforming bacterial strains are classified in the Proteobacteria phylum, and strains of Alphaproteobacteria, including those of Rhodospirillum rubrum (13), Rhodobacter capsulatus (63), Rhizobium sp. (64), and Agrobacterium sp. (65) have been used in culture-based studies to investigate bacterial Se reduction mechanisms.

Many of the Alphaproteobacteria and Actinobacteria sequences matched known taxa related to N cycling, including Rhizobiales and Bradyrhizobiaceae (Alphaproteobacteria) and Frankiales and Micrococcales (Actinobacteria). Many known N-cycling bacteria are also able to reduce Se oxyanions, either aerobically or anaerobically (61, 64), and at least one Rhizobiales (Agrobacterium tumefaciens) has been isolated from nearby reclaimed mines in the Phosphate Patch and shown to reduce selenate (15). Some organisms can reduce selenate via nitrate reductases (66), and several nitrate-reducing OTUs were seen to increase significantly in a saturated soil experiment following selenate amendment (35). While this suggests that there is a shared enzymatic mechanism for nitrate and selenate reduction in certain bacterial populations, it is also known that there are alternative pathways for Se reduction that remain poorly understood (17).

There were no differences at the phylum level of the fungal community in the different sites or years, and soils were dominated by Ascomycota (∼70%) and Basidiomycota (12.5%), as is typical of grasslands and shrublands (67). Within the Ascomycetes, two families (Trichocomaceae and Pezizomycotina [incertae sedis]) had significantly increased relative abundance in high-Se soils compared to low-Se soils, and all significant OTUs (97% similarity) in the high-Se soils were members of the Ascomycota phylum. The vast majority of fungal cultures known to reduce Se oxyanions are members of the Ascomycota phylum (17, 36, 68), which is likely representative of the high metal tolerance (69) and metal redox capabilities (70, 71) exhibited by many members of the phylum.

Interestingly, the dominant Basidiomycota showed a substantially different pattern than the Ascomycetes in high- and low-Se soils. Of the top 10 most abundant Basidiomycota families present in the low-Se soils (all with relative abundances greater than 1%), five families were reduced to less than 0.5% in the high-Se soils (three were 0.01% or less). This pattern was not evident with regard to mining history or year (i.e., all top 10 Basidiomycota families were present in both mined and nonmined sites with similar abundances), indicating that the unique conditions of the high-Se soils were specifically associated with the absence of these Basidiomycota families. All five families belong to the same order, Agaricales, a well-known fungal order that forms gilled mushroom fruiting bodies and has a capacity for both cellulose and lignin degradation (72, 73). There is limited information regarding metal(loid) tolerance of Basidiomycota fungi in contaminated soils. Some Agaricales families have been shown to tolerate and accumulate high concentrations of metals (74, 75), while others have shown decreased soil colonization (76) and inhibited growth (77) in the presence of elevated metal concentrations. Basidiomycota enzymatic activity specifically related to the breakdown of lignin and/or cellulose has also been shown to be negatively impacted by metal(loid) contaminants in soils (76, 78), suggesting that elevated metal(loid) concentrations may alter C cycling in reclaimed mine soils.

Studies of mine reclamation have commonly focused on physicochemical soil characteristics (e.g., organic C concentration, nutrient content, and aggregate stability) or vegetation characteristics (79, 80), which often recover faster than microbial communities (81). This may mean that sustained differences in nutrient cycling and resulting impacts of those altered elemental cycles may persist long after physicochemical parameters suggest complete site recovery. In the current study, the loss of several C-degrading Basidiomycota families in high-Se soils may ultimately influence C decomposition and storage in high-Se soils. Additionally, long-term impacts of altered fungal communities may inhibit nutrient release and availability, thereby feeding back to limitations on vegetative and microbial productivity (82). Future work, however, is required to explicitly test the impacts of Se on basidiomycete diversity and enzymatic activity, as well as the associated impacts of the loss of these particular families on C accumulation or loss from these soils.

### Concluding remarks.

Using both OTU-based diversity assessments and taxonomic patterns of microbial communities in reclaimed soils, high-throughput DNA sequencing offers opportunities to assess reclamation status and the relationship of communities to geochemical (e.g., pollutant concentration and organic carbon content) and physical parameters, as well as to identify strains with potential for bioremediation of geochemically complex sites (83). Though traditional assessment methods focus on physicochemical measures to determine reclamation status, microbial communities have recently been recognized as important indicators of mine reclamation success, which may lag behind physicochemical measurements. The mine soils used in this study were reclaimed 20 to 30 years ago, though the bacterial and fungal communities in mined sites remain distinct from those in unmined sites, and of diminished diversity. Alterations of this magnitude have the potential to influence C cycling in contaminated sites via changes to the lignocellulose-degrading community, with associated negative impacts on plant and microbial productivity in the reclaimed mine soils. DNA sequences from our sites were dominated by phylotypes involved in N cycling, and many known N-cycling bacteria are also known to reduce Se oxyanions; therefore, we hypothesize that Se and N biogeochemical cycles may be linked in these aerobic soil ecosystems. Future work is needed to understand the long-term site-specific impacts in this area. We have also used nonparametric statistical analysis of abundant OTUs to identify a constrained number of OTUs (10 bacterial and 5 fungal) that were unique to high-Se soils. As the genetic mechanisms for aerobic Se reduction are not well known, data of this type offer an opportunity to couple culture-independent data with culturing to isolate effective Se bioremediators already adapted to the complex geochemical conditions present on the Champ and Mountain Fuel sites.

## MATERIALS AND METHODS

### Field site and sampling.

Soil samples were obtained from the Champ and Mountain Fuel Mines within the upper Dry Valley of Caribou County in the Southeastern Idaho Phosphate Mining District, ID. These mines overlie the Phosphoria Formation, containing phosphate ore (phosphorite) sandwiched between Se-rich shale units (30). Following open-pit phosphate mining, the two mines were reclaimed in a similar fashion 20 to 30 years ago by covering leftover rock dumps with waste rock, including Se-enriched center waste shale, and were seeded with brome grass (Bromus marginatus) and alfalfa (Medicago sativa) as primary species. The center waste shale is known to be highly enriched with Se, with concentrations up to 1,000 ppm, which is significantly greater than the global average for shale (84). Two separate 1-week field campaigns were conducted during June 2015 and June 2016 to collect soils for microbial community and geochemical analysis. Soil samples from six locations within the Champ Mine and seven locations within the Mountain Fuel Mine were collected. At each location, surface soils (∼0 to 10 cm) from an area of approximately 25 cm by 25 cm were collected and composited, after which three individual subsamples were collected. A duplicate 25-cm-by-25-cm area was also collected at each location, composited, and subsampled in triplicate to ensure that the heterogeneity within the microbial community at each location was adequately captured. Locations were selected across the extent of both mines to encompass a range of geochemical (particularly Se concentration) conditions in the mine soils, with expected Se concentrations ranging from 1 to 100 mg · kg^−1^ Se (85, 86). Additionally, one reference area for each mine was sampled in a fashion similar to that for the mined sites. In both cases, these reference areas were located in the same spatial area as the mined sites; however, they were outside the mining and reclamation footprint. Both reference sites had more diverse plant communities than their mined counterparts, though the dominant mine vegetation (brome grass and alfalfa) was also present on the reference areas. Of particular note was the presence of sagebrush (Artemisia tridentata) on the Champ Mine reference area and numerous flowering plants on the Mountain Fuel Mine reference area. Once collected, soil samples for microbial community analysis were immediately flash-frozen in a dry ice-ethanol bath in the field and stored on dry ice during transportation to the laboratory, where they were stored at −80°C until processing. Duplicate samples were also collected from each sampling location for soil geochemical analysis. These samples were collected in 50-ml Falcon tubes and stored at 4°C during transportation to the laboratory, at which point they were air-dried in the laboratory, sieved to <2 mm, and stored at room temperature prior to analysis.

### Soil geochemical analysis.

Subsamples (approximately 10 g) of air-dried and sieved (<2 mm) soils were ground for homogenization using a Spex ball mill and acid-digested via microwave digestion using a standard HF digestion protocol (87) before analysis for As, Cd, Fe, Mn, Ni, Se, and Zn levels via inductively coupled plasma mass spectrometry (ICP-MS; Thermo Scientific X2). Additional subsamples were ground to <100 μm and analyzed for organic C and total N levels via dry combustion elemental analysis (vario PYROcube; Elementar). Prior to analysis, samples were pretreated to remove carbonates using 6% H_2_SO_3_ until all reaction had ceased and were dried at 105°C to remove residual water. Soil pH was measured in a 1:2 slurry with deionized water using a pH meter (FEP20; Mettler Toledo).

### DNA sequencing.

DNA was extracted from each of the triplicate samples collected from each sampling location using the FastDNA Spin kit for soil (MP Biomedicals), with the following modifications. Polyadenosine [poly(A); 200 μg per sample] was added to the lysis buffer to reduce inhibition by metal(loid) cations (88). Two homogenization steps on the FastPrep instrument (MP Biomedicals) were carried out, with a 5-min incubation on ice between them. The initial centrifugation step to remove soil and cell debris was extended to 15 min, and the binding matrix incubation was extended to 10 min. Elution was carried out by resuspending the binding matrix in 100 μl nuclease-free sterile water and incubating at 55°C. Extracts were quantified using the Qubit double-stranded DNA broad-range (BR) assay kit (Life Technologies) with a Qubit 2.0 fluorometer, and those exceeding 100 ng · μl^−1^ were diluted to that concentration. Extracts from the triplicate samples per location duplicate were then pooled into a single sample and used in DNA amplification and sequencing at the University of Minnesota Genomics Center using a dual-indexing approach modified from the Earth Microbiome Project protocols (89). Additional negative controls were also extracted in triplicate using identical procedures to assess any microbial taxa contained within the extraction kits themselves. The following three target regions were amplified and sequenced from all samples: (i) bacterial and archaeal 16S rRNA gene region using V4 hypervariable region primers 505F and 806R (89), (ii) fungal internal transcribed spacer region 1 (ITS1) using ITS1F and ITS2R primers (90), and (iii) fungal internal transcribed spacer region 2 (ITS2) using primers ITS4 and 5.8R′ (90). After amplification, DNA samples were then diluted (1:100 or 1:1,000 for V4 and 1:25 or 1:125 for ITS1/ITS2) to reduce amplification inhibition and subjected to paired-end sequencing with a MiSeq 600 cycle version 3 kit (Illumina, San Diego, CA).

Raw DNA sequences were processed using the mothur software package (version 1.39.5) (91), according to the protocols described by Kozich et al. (92). For the 16S region, this entailed merging paired-end reads into contigs, quality screening the contigs, aligning sequences to the SILVA 16S rRNA sequence database, and screening and removing chimeras. Bacterial and archaeal sequences were further cleaned by classifying against the Silva reference database in mothur using the method developed by Wang et al. (93). Processed sequences were then clustered into operational taxonomic units (OTUs) using a 97% sequence similarity cutoff (OTU_0.03_) with the OptiClust algorithm in mothur (94) and assigned a consensus taxonomy. For full-length assembled fungal ITS1 sequences, ∼50% of the sequences were dropped due to the inability to form contigs with >25 bp overlap. As a result, only ITS1 forward reads were used to retain fungal groups with long ITS1 regions (i.e., the ITS1 region is too long to form contigs with >25 bp overlap). In this case, all sequences were trimmed to the same length (279 bp), and low-quality (*Q* score < 25) reads were removed. Fungal sequences were classified against the UNITE+INSD database (version 7.1) (95) and clustered into OTUs with a 97% sequence similarity cutoff using vsearch with the abundance-based method implemented in mothur (90). To eliminate the potential inclusion of artifacts from rare OTUs (96), only OTUs that consisted of more than 10 sequences were retained for analysis. Full-length contigs of the fungal ITS2 region were also assembled. Classification, clustering, and removal of rare OTUs for the ITS2 sequences were performed as discussed for the ITS1 region. The results from ITS1 and ITS2 sequences were highly similar; therefore, we have followed the remaining analysis through using the ITS1 region only. For both bacteria and fungi, the number of sequences of each OTU present in the negative controls (PCR and DNA extractions) were manually subtracted from the sequence abundance of that OTU in the experimental samples using R version 3.4.2 (97). This approach allowed us to maintain ecologically relevant OTUs, while still accounting for contamination and limiting noise associated with the sequence data sets (98).

### Statistical analysis.

Data were checked for assumptions of normality and log-transformed where necessary. Alpha- and beta-diversity metrics, all statistical comparisons, and plotting were carried out in R (97). The vegan package version 2.4-4 (99) was used to calculate indices of α-diversity (observed OTU richness, inverse Simpson, and Shannon indices), as well as principal-coordinate analysis (PCoA; β-diversity). Prior to calculating OTU richness, total bacterial and fungal OTU abundances were randomly subsampled according to the library with the lowest sequence coverage (1,561 bacterial and 4,616 fungal sequences). β-Diversity was calculated by the Bray-Curtis dissimilarity metric, using Hellinger-transformed OTU abundances (100). These pairwise dissimilarities were first used to visually determine variation in community composition using PCoA. Permutational multivariate analysis of variance (PERMANOVA [101]) was used to further examine the significance of compositional differences of bacterial and fungal communities among samples that differed in relation to site history (mined or not mined), sample location (mined and reference sites in either the Champ Mine area or Mountain Fuel Mine area), year collected (2015 or 2016), or Se abundance (<30 ppm Se or >30 ppm Se). In the case of a significant *P* value in PERMANOVA, a distance-based test for homogeneity of multivariate dispersions (PERMDISP [102]) was used. PERMDISP uses the analysis of variance (ANOVA) *F*-statistic to assess whether observed shifts were driven by multivariate heterogeneity among groups by comparing differences in the distance from individual observations to their group centroid (median). Significant differences among groups are tested through permutation of least-squares residuals. Both PERMANOVA and PERMDISP were run based on Hellinger-transformed Bray-Curtis dissimilarities and 999 permutations. PERMANOVA and PERMDISP were run using functions ‘adonis’ and ‘betadisper,’ respectively, in the R vegan package. Finally, the function ‘envfit’ was used to create a secondary matrix of geochemical factors (pH and organic C, N, Fe, Mn, Se concentrations), which could be related to the relative ordination of Champ and Mountain Fuel sites. For this analysis, a nonparametric Spearman's correlation coefficient was used to determine correlations between microbial community structure and soil geochemistry. As with the PERMANOVA and PERMDISP analyses, the correlations of geochemical parameters and ordination were run based on 999 permutations.

The relationship between Se concentration and α-diversity metrics (observed OTU richness, inverse Simpson index, and Shannon index) was measured using Pearson correlation calculated in R with the ‘lm’ function. Other geochemical components (pH and organic C, N, Fe, and Mn concentrations) were assessed similarly for their correlation with α-diversity via the ‘lm’ function. To test for bacterial or fungal OTU enrichment in low- and high-Se soils, the nonparametric Mann-Whitney U test with *P* values corrected for multiple comparisons by false-discovery rate method was used.

### Accession number(s).

All sequence data were deposited, with MIMARKS-compliant metadata, in the NCBI Sequence Read Archive under BioProject number PRJNA450848, with BioSample numbers SAMN08949802 to SAMN08949876.

## Supplementary Material

Supplemental material
