# Supplementary material for: Persistent Bacterial and Fungal Community Shifts Exhibited in Selenium-Contaminated Reclaimed Mine Soils
Source: Appl Environ Microbiol. 2018 Aug 1;84(16):e01394-18. doi: 10.1128/AEM.01394-18 (PMC6070768; doi:10.1128/AEM.01394-18)
Supplement: Supplemental material [file supp_84_16_e01394-18__index.html]

Supplemental material 

# Persistent Bacterial and Fungal Community Shifts Exhibited in Selenium-Contaminated Reclaimed Mine Soils

## Supplemental material

- Supplemental file 1 -

  Soil parameters (Table S1); results of linear models (Table S2); Spearman’s correlation coefficients (Table S3); relative abundances by one-way analysis of variance (Tables S4 and S7) and nonparametric Mann-Whitney U test (Tables S6 and S11); two-way analysis of variance results (Tables S5 and S8 to S10); map and location images (Fig. S1); correlation data (Fig. S2); relative abundances (Fig. S3); taxonomic affiliations (Fig. S4); most abundant bacterial orders in high- and low-Se soils (Fig. S5).

  PDF, 6.8M
