# Supplementary material for: Persistent Bacterial and Fungal Community Shifts Exhibited in Selenium-Contaminated Reclaimed Mine Soils
Source: Appl Environ Microbiol. 2018 Aug 1;84(16):e01394-18. doi: 10.1128/AEM.01394-18 (PMC6070768; doi:10.1128/AEM.01394-18)
Supplement: Supplemental material [file AEM.01394-18_zam016188679s1.pdf]

Supplemental Information

Persistent bacterial and fungal community shifts exhibited in selenium-contaminated reclaimed mine soils

Carla E. Rosenfeld[1,2], Bruce R. James[3], and Cara Santelli[1,2,*]

[1]Department of Earth Sciences, University of Minnesota – Twin Cities, Minneapolis, MN USA

[2]BioTechnology Institute, University of Minnesota – Twin Cities, St. Paul, MN USA

[3]Department of Environmental Science and Technology, University of Maryland, College Park, MD USA

[*]Corresponding author information: 1479 Gortner Ave, Suite 140, St. Paul, MN 55108; santelli@umn.edu; phone: 612-624-9760; fax: 612-625-5780

Table S1: pH, Se concentration, organic C and total N content in soils collected from Champ and Mountain Fuel Mine areas during 2015 and 2016.

Mine	Location	Year	pH	Se (mg/kg)	Organic C (g/kg)	N (g/kg)
Champ Mine (CM)	BP1	2015	8.1	16.0	19.4	1.8
		2016	8.1	61.3	73.2	7.3
	ED1	2015	7.3	5.4	34.0	2.9
		2016	6.8	6.4	26.8	3.0
	ED2	2015	7.7	4.6	31.5	2.9
		2016	7.5	4.5	33.8	3.3
	ED3	2015	6.6	64.0	55.8	7.3
		2016	7.2	50.7	43.1	6.9
	ED4	2015	7.6	56.6	41.5	4.7
		2016	7.5	69.0	33.0	5.5
	ED5	2015	7.3	9.0	26.4	2.2
		2016	8.1	77.1	66.0	5.9
	Reference	2015	6.4	1.7	40.9	3.4
		2016	6.7	0.9	34.5	3.3
Mountain Fuel Mine (MFM)	NPD1	2015	6.9	12.3	29.8	6.2
		2016	7.3	7.9	12.0	6.4
	NPD2	2015	7.1	7.1	15.3	1.3
		2016	7.7	5.0	9.8	1.0
	NPD3	2015	7.9	4.9	8.5	0.5
		2016	7.5	38.3	22.8	2.9
	SD1	2015	6.5	38.9	43.3	6.9
		2016	7.0	19.0	21.7	3.7
	VD1	2015	7.5	7.3	27.0	2.0
		2016	8.0	4.5	11.8	1.1
	VD2	2015	7.7	4.3	14.8	0.9
		2016	7.5	3.3	7.5	1.4
	VD3	2015	6.8	7.2	15.0	2.2
		2016	7.8	7.6	17.8	2.3
	Reference	2015	7.1	1.7	40.7	3.2
		2016	7.2	0.8	40.2	3.6

Notes:
Data represent averages calculated from duplicate samples collected in each year. White shaded rows show data collected in 2015. Grey shaded rows show data collected in 2016.

Table S2: Results of linear models comparing three α-diversity metrics (Inverse Simpson, OTU Richness, and Shannon) to various geochemical parameters measured in the soils.

	**Inverse Simpson**			**Richness**			**Shannon**		
	slope	R^2_{adj}	p-value	slope	R^2_{adj}	p-value	slope	R^2_{adj}	p-value
Bacteria									
pH	-462	0.13	0.001	-66.57	0.18	0.00008	-0.253	0.07	0.01
Organic C	-12.67	0.001	0.78	-3.17	0.001	0.57	-0.018	0.001	0.58
N	-818.76	0.06	0.03	-118.03	0.08	0.009	-0.54	0.05	0.03
log(Fe)	230.74	0.009	0.56	6.7	0.001	0.89	0.062	0.001	0.82
log(Mn)	322.56	0.15	0.0003	43.13	0.19	0.00006	0.21	0.13	0.0008
Fungi									
pH	-58.8	0.075	0.01	-57.01	0.07	0.01	-0.23	0.06	0.02
Organic C	-1.67	0.001	0.8	-4.55	0.006	0.54	-0.029	0.01	0.35
N	-125.58	0.05	0.03	-147.29	0.08	0.01	-0.7	0.1	0.005
log(Fe)	149.2	0.06	0.02	148.8	0.07	0.02	0.33	0.008	0.22
log(Mn)	65.66	0.27	$2x10^{-6}$	69.39	0.3	<0.00001	0.27	0.27	$2x10^{-6}$

Notes:
P-values <0.05 are considered statistically significant.

Table S3: Spearman's correlation coefficients (R^2) for envfit results comparing geochemical factors with bacterial and fungal OTU ordinations.

	R^2	P
Bacteria		
pH	0.52	0.001
Fe	0.29	0.001
Mn	0.21	0.003
organic C	0.11	0.03
N	0.10	0.03
Se	0.06	0.13
Fungi		
pH	0.48	0.001
Fe	0.27	0.001
Mn	0.22	0.001
organic C	0.11	0.03
N	0.04	0.27
Se	0.07	0.11

Notes:
P-values <0.05 are considered statistically significant.

Table S4: Mean relative abundances and associated adjusted p-value from one-way analysis of variance for top bacterial phyla (or class, in the case of proteobacteria) in low and high Se soils.

Bacteria Phylum*	Mean Relative Abundance (%)		$p_{adjusted}$
	low Se	high Se	
Actinobacteria	26.0	28.0	0.005
Bacteroidetes	10.8	9.8	0.13
Alphaproteobacteria	10.7	12.1	0.28
Acidobacteria	9.9	9.4	0.15
Chloroflexi	8.2	8.4	0.34
Verrucomicrobia	6.7	5.7	0.12
Planctomycetes	5.8	6.2	0.34
Deltaproteobacteria	4.4	4.1	0.13
Gemmatimonadetes	3.9	5.7	0.0002
Betaproteobacteria	3.8	3.4	0.34
Firmicutes	3.1	0.6	0.07
Gammaproteobacteria	2.6	2.1	0.12

Notes:
Adjusted p-values ($p_{adjusted}$) <0.05 are considered statistically significant.
*Remaining bacterial phyla with <1% mean relative abundance of all bacterial sequences were: Aminicenantes, Armatimonadetes, Atribacteria, BJ-169, BRC1, Candidatus Berkelbacteria, Chlamydiae, Chlorobi, Cyanobacteria, Deinococcus-Thermus, Elusimicrobia, FBP, FCPU426, Fibrobacteres, Fusobacteria, GAL15, Gracilibacteria, Hydrogenedentes, Ignavibacteriae, Latescibacteria, Microgenomates, Nitrospirae, Omnitrophica, Parcubacteria, Peregrinibacteria, RBG-1 Zixibacteria, Saccharibacteria, SBR1093, Spirochaetae, SR1 Absconditabacteria, Synergistetes, Tectomicrobia, Tenericutes, TM6 Dependentiae, WS2, and WWE3.

Table S5: Two-way analysis of variance table for most abundant bacterial phyla comparing mined and reference sites and sampling year.

Bacterial Phylum	Factor	$p_{adjusted}$
Actinobacteria	mine history	0.12
	year	2.02E-32
	mine history x year	0.12
Alphaproteobacteria	mine history	0.002
	year	0.38
	mine history x year	0.73
Bacteroidetes	mine history	1.31E-11
	year	4.98E-37
	mine history x year	0.10
Acidobacteria	mine history	2.87E-05
	year	0.71
	mine history x year	0.002
Chloroflexi	mine history	0.16
	year	0.71
	mine history x year	0.71
Verrucomicrobia	mine history	3.62E-10
	year	4.28E-08
	mine history x year	0.12
Planctomycetes	mine history	4.66E-07
	year	2.54E-66
	mine history x year	0.67
Deltaproteobacteria	mine history	0.12
	year	1.20E-16
	mine history x year	0.71
Gemmatimonadetes	mine history	0.98
	year	0.89
	mine history x year	0.98
Betaproteobacteria	mine history	0.002
	year	0.16
	mine history x year	0.75
Firmicutes	mine history	0.42
	year	0.001
	mine history x year	0.16
Gammaproteobacteria	mine history	0.001
	year	0.00002
	mine history x year	0.12

Notes:
Mine history factor compared samples collected from mined sites to samples collected from reference (non-mined) sites.
Adjusted p-values <0.05 are considered statistically significant.

Table S6: Mean relative abundances and associated adjusted p-value for OTUs with statistically significant ($p_{adjusted}<0.05$) relative abundances in high and low Se soils, as tested using nonparametric Mann-Whitney U test.

Phylum	OTU	Consensus Taxonomy*	Mean Relative Abundance (%)		$p_{adjusted}$
			low Se	high Se	
Acidobacteria	OTU 39	family Blastocatellaceae	0.50	0.05	0.003
Acidobacteria	OTU 48	phylum Acidobacteria	0.35	0.19	0.033
Acidobacteria	OTU 58	phylum Acidobacteria	0.31	0.17	0.031
Acidobacteria	OTU 93	family Blastocatellaceae	0.18	0.02	0.001
Acidobacteria	OTU 107	phylum Acidobacteria	0.15	0.09	0.029
Acidobacteria	OTU 155	family Blastocatellaceae	0.13	0.02	0.033
Actinobacteria	OTU 11	family Micrococcaceae	0.35	0.04	0.005
Actinobacteria	OTU 55	family Micromonosporaceae	0.24	0.44	0.029
Actinobacteria	OTU 98	genus *Kribbella sp.*	0.10	0.20	0.029
Actinobacteria	OTU 102	genus *Amycolatopsis sp.*	0.10	0.48	0.004
Actinobacteria	OTU 118	genus *Nocardioides sp.*	0.13	0.26	0.033
Actinobacteria	OTU 124	order Solirubrobacterales	0.17	0.04	0.031
Actinobacteria	OTU 129	order Solirubrobacterales	0.12	0.20	0.048
Actinobacteria	OTU 168	genus *Nocardioides sp.*	0.08	0.20	0.031
Actinobacteria	OTU 172	genus *Lentzea sp.*	0.08	0.29	0.004
Bacteroidetes	OTU 110	genus *Segetibacter sp.*	0.09	0.24	0.033
Chloroflexi	OTU 41	phylum Chloroflexi	0.45	0.18	0.014
Chloroflexi	OTU 119	family Anaerolineaceae	0.16	0.06	0.012
Firmicutes	OTU 14	order Bacillales	1.07	0.27	0.014
Firmicutes	OTU 23	family Planococcaceae	0.69	0.18	0.005
Firmicutes	OTU 97	genus *Domibacillus sp.*	0.27	0.04	0.004
Gemmatimonadetes	OTU 30	family Gemmatimonadaceae	0.33	1.06	0.040
Gemmatimonadetes	OTU 82	genus *Gemmatimonas sp.*	0.06	0.26	0.029
Verrucomicrobia	OTU 64	order Chthoniobacterales	0.33	0.01	0.040
Verrucomicrobia	OTU 288	order Chthoniobacterales	0.14	0.01	0.004

Notes:
*Consensus taxonomy assigned at 80% confidence cutoff.

Table S7: Relative abundances and associated adjusted p-value from one-way analysis of variance for all sequences classified at the fungal phylum level in low and high Se soils.

Fungal Phylum	Mean Relative Abundance (%)		$p_{adjusted}$
	low Se	high Se	
Ascomycota	68.9	69.6	0.98
Basidiomycota	11.9	14.5	0.95
Chytridiomycota	2.8	1.2	0.95
Glomeromycota	1.3	1.3	0.98
Rozellomycota	0.01	0.05	0.77
Zygomycota	5.2	4.3	0.95

Notes:
Adjusted p-values <0.05 are considered statistically significant.

Table S8: Two-way analysis of variance table for fungal phyla comparing mined and reference sites and sampling year.

Fungal Phylum	Factor	$p_{adjusted}$
Ascomycota	mine history	0.49
	year	0.49
	mine history x year	0.85
Basidiomycota	mine history	0.72
	year	0.49
	mine history x year	0.85
Chytridiomycota	mine history	0.49
	year	0.49
	mine history x year	0.85
Glomeromycota	mine history	0.49
	year	0.49
	mine history x year	0.72
Rozellomycota	mine history	0.49
	year	0.85
	mine history x year	0.85
Zygomycota	mine history	0.0002
	year	0.49
	mine history x year	0.85

Notes:
Mine history factor compared samples collected from mined sites to samples collected from reference (non-mined) sites.
Adjusted p-values <0.05 are considered statistically significant.

Table S9: Two-way analysis of variance table for fungal families in the Ascoymycota phylum, comparing mined and reference sites and sampling year.

Family	Factor	$p_{adjusted}$
**Ascomycota**		
Pleosporales (*incertae sedis*)	mine history	0.19
	year	0.66
	mine history x year	0.94
Trichocomaceae	mine history	0.88
	year	0.27
	mine history x year	0.95
Lasiosphaeriaceae	mine history	0.56
	year	0.77
	mine history x year	0.94
Helotiales (*incertae sedis*)	mine history	0.19
	year	0.77
	mine history x year	0.36
Pezizomycotina (*incertae sedis*)	mine history	0.38
	year	0.36
	mine history x year	0.23
Sporormiaceae	mine history	0.38
	year	0.19
	mine history x year	0.27
Mycosphaerellaceae	mine history	0.38
	year	0.27
	mine history x year	0.56
Nectriaceae	mine history	0.25
	year	0.25
	mine history x year	0.77
Helotiaceae	mine history	0.38
	year	0.19
	mine history x year	0.77
Phaeosphaeriaceae	mine history	0.45
	year	0.27
	mine history x year	0.74

Notes:
Mine history factor compared samples collected from mined sites to samples collected from reference (non-mined) sites.
Adjusted p-values <0.05 are considered statistically significant.

Table S10: Two-way analysis of variance table for fungal families in the Basidiomycota phylum, comparing mined and reference sites and sampling year.

Family	Factor	$p_{adjusted}$
**Basidiomycota**		
Ceratobasidiaceae	mine history	0.49
	year	0.94
	mine history x year	0.94
Entolomataceae	mine history	0.65
	year	0.65
	mine history x year	0.88
Tremellales (*incertae sedis*)	mine history	0.002
	year	0.88
	mine history x year	0.72
Hydnodontaceae	mine history	0.85
	year	0.73
	mine history x year	0.88
Bolbitiaceae	mine history	0.88
	year	0.65
	mine history x year	0.88
Piskurozymaceae	mine history	0.88
	year	0.88
	mine history x year	0.49
Clavariaceae	mine history	0.65
	year	0.44
	mine history x year	0.88
Lyophyllaceae	mine history	0.94
	year	0.88
	mine history x year	0.65
Mycenaceae	mine history	0.002
	year	0.65
	mine history x year	0.44
Agaricaceae	mine history	0.76
	year	0.85
	mine history x year	0.65

Notes:
Mine history factor compared samples collected from mined sites to samples collected from reference (non-mined) sites.
Adjusted p-values <0.05 are considered statistically significant.

Table S11: Mean relative abundances and associated adjusted p-value for OTUs with statistically significant ($p_{adjusted}$<0.05) relative abundances in high and low Se soils, as tested using nonparametric Mann-Whitney U test.

Phylum	OTU	Consensus Taxonomy*	Mean Relative Abundance (%)		$p_{adjusted}$
			low Se	high Se	
Chytridiomycota	OTU 7	genus *Olpidium sp.*	0.57	0.00	0.022
Ascomycota	OTU 17	genus *Paraboeremia sp.*	0.74	0.08	0.039
Ascomycota	OTU 32	phylum Ascomycota	0.67	0.00	0.022
Ascomycota	OTU 45	genus *Podospora sp.*	0.37	0.00	0.039
Ascomycota	OTU 62	genus *Phaeomycocentrospora sp.*	0.16	0.57	0.039
Ascomycota	OTU 96	phylum Ascomycota	0.10	0.46	0.039
Ascomycota	OTU 97	genus *Chaetomium sp.*	0.25	0.00	0.028
Ascomycota	OTU 113	genus *Purpureocillium sp.*	0.08	0.55	0.039
Ascomycota	OTU 122	genus *Penicillium sp.*	0.09	0.29	0.039
Ascomycota	OTU 136	genus *Humicola sp.*	0.20	0.00	0.046
Ascomycota	OTU 176	genus *Paraphoma sp.*	0.17	0.02	0.001
Ascomycota	OTU 558	genus *Gorgomyces sp.*	0.00	0.49	0.00002

Notes:
*Consensus taxonomy was assigned with 80% confidence cutoff.

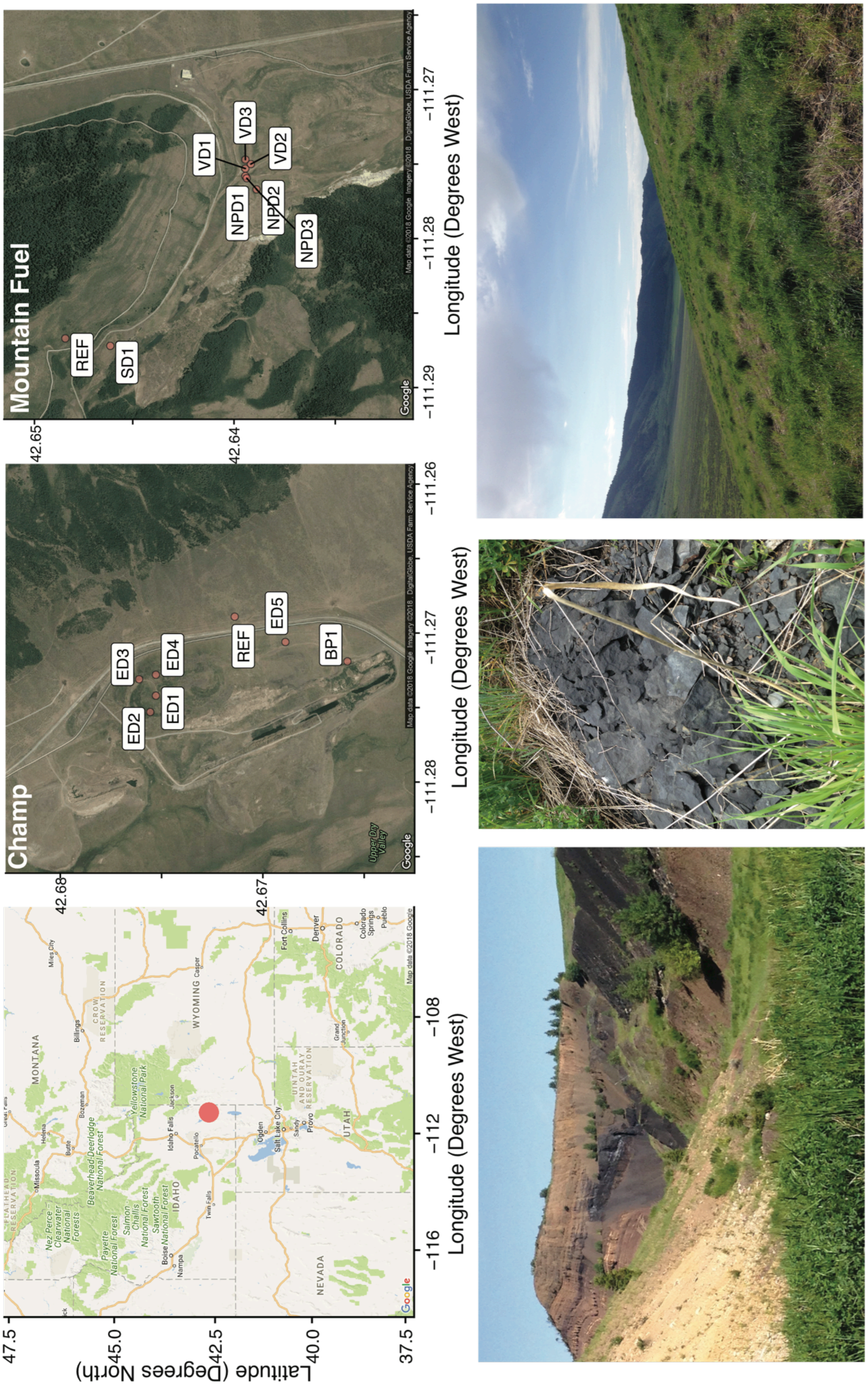
Champ
Mountain Fuel
Latitude (Degrees North)
47.5
45.0
42.5
40.0
37.5
−116
−112
−108
Longitude (Degrees West)
42.68
42.67
−111.28
−111.27
−111.26
ED1
ED2
ED3
ED4
ED5
REF
BP1
42.65
42.64
−111.29
−111.28
−111.27
REF
SD1
NPD1
NPD2
NPD3
VD1
VD2
VD3

Fig S1: Overview map of the Idaho region (A) with sampling area approximately located at the position of the red dot. Map of sampling locations (red dots) within the Champ Mine (CM; B) and Mountain Fuel Mine (MFM; C). Photo of shale placement on soil surface at location CM-ED4 (D), and photo taken at CM-ED3 depicting typical vegetation density found throughout CM and MFM sites (E). During both years, duplicate samples were collected at each location from separate holes for DNA and geochemical analysis.

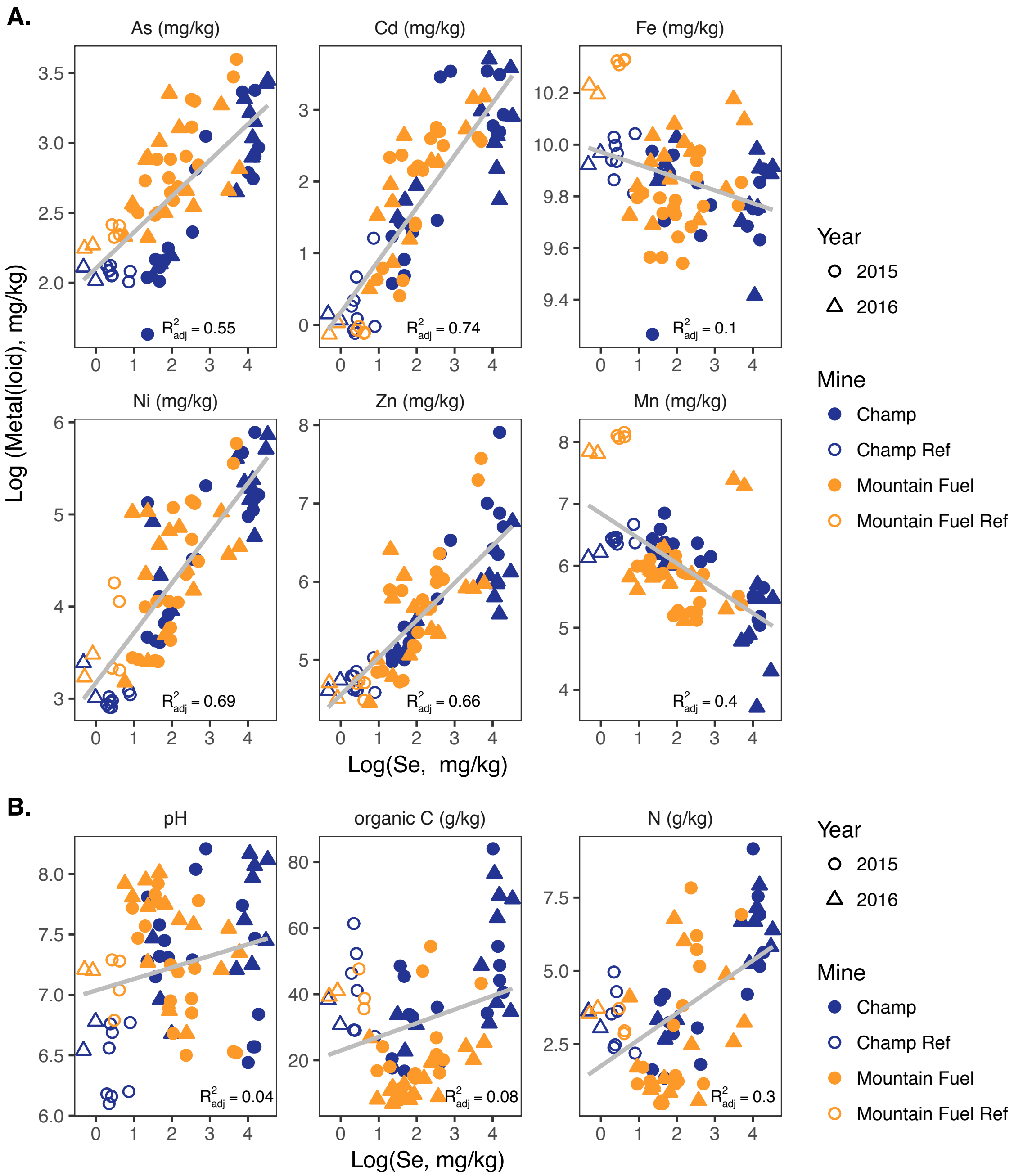

Fig S2: Correlations of log-transformed Se concentration with log-transformed geochemical constituents (As, Cd, Fe, Ni, Zn, and Mn) (A) and pH, organic C, and total N (B) in soils in the Champ Mine (dark blue, closed symbols), Champ Reference (dark blue, open symbols), Mountain Fuel Mine (orange, closed symbols), and Mountain Fuel Reference (orange, open symbols). All regressions were statistically significant ($p<0.05$), with adjusted R^2 (R^2_{adj}) values listed in the bottom of each panel. Each data point represents an individual sample taken in either 2015 (circles) or 2016 (triangles).

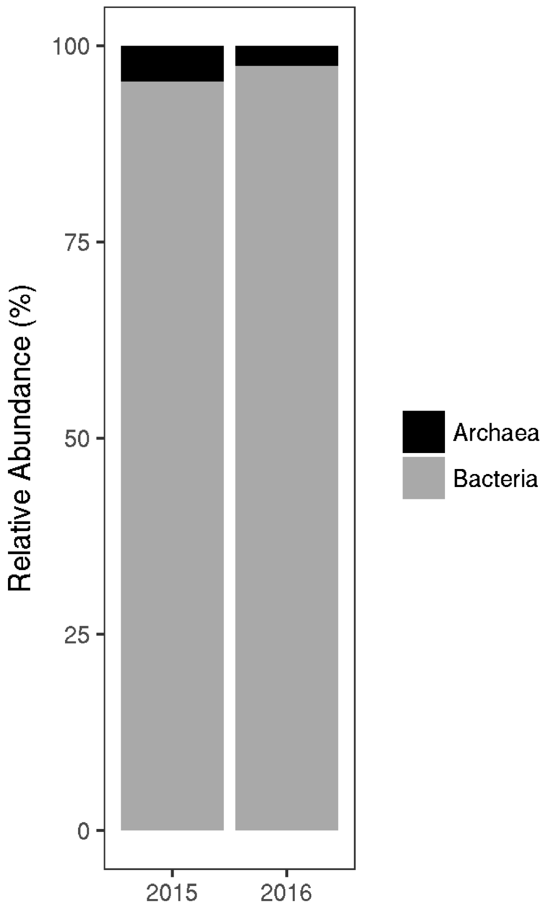

Fig S3: Relative abundance of archaeal (black bars) and bacterial sequences (grey bars) in soils during 2015 and 2016.

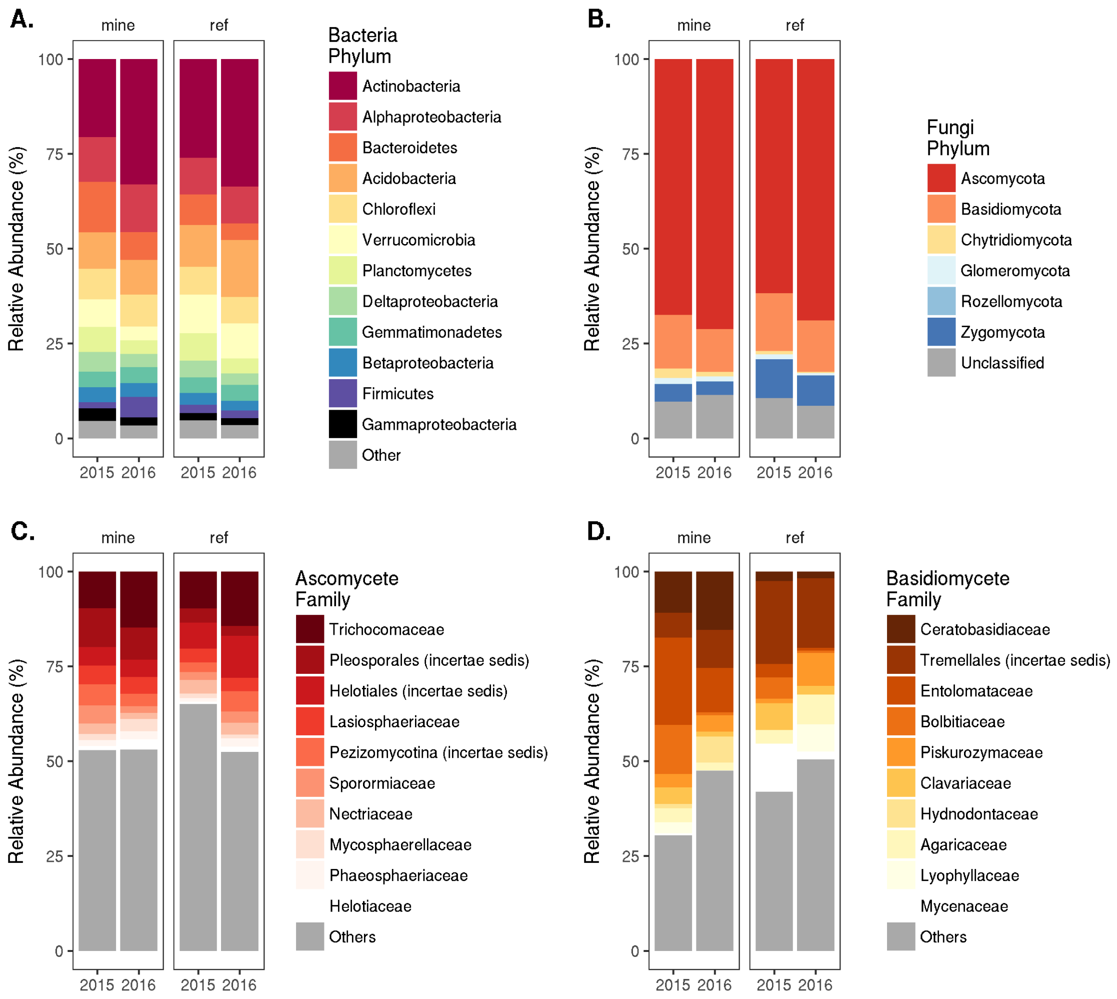

Fig S4: Taxonomic affiliation of bacterial (phylum level, A) and fungal sequences (phylum level, B, and family level, C & D) in mined and reference areas during 2015 and 2016.

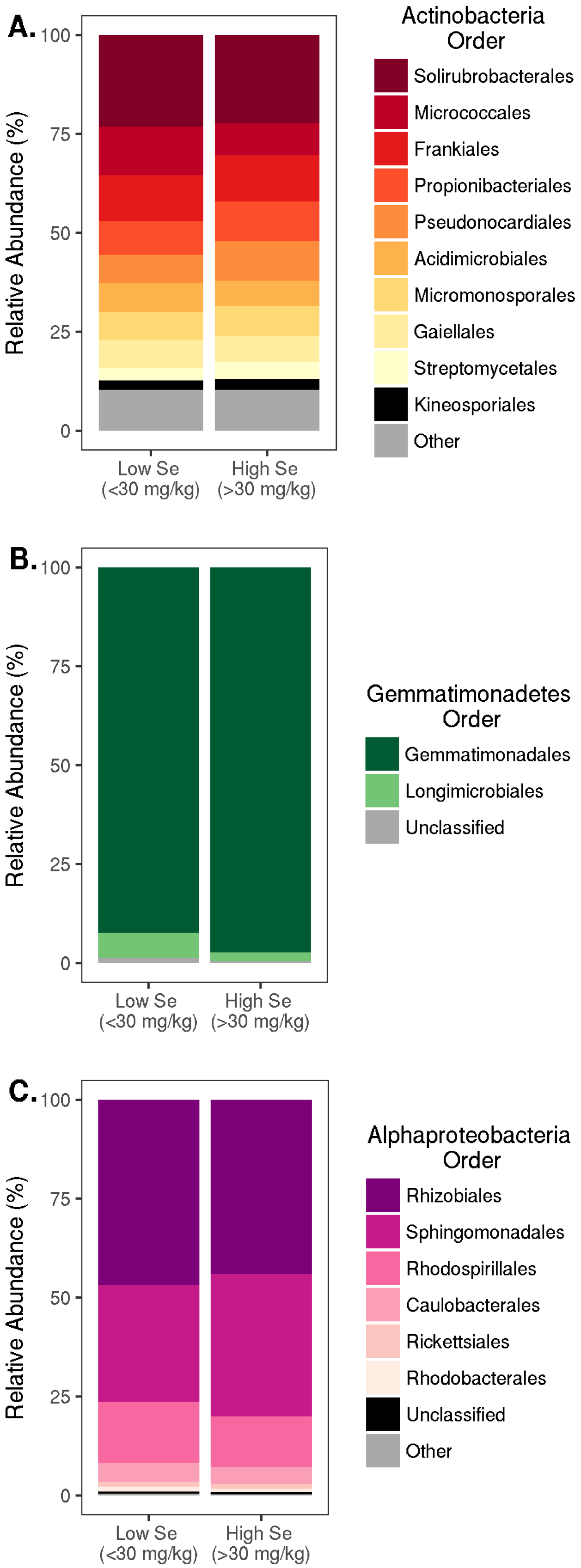

Fig S5: Top most abundant bacterial orders in high and low Se soils in Actinobacteria (A) and Gemmatimonadetes (B) phyla and Alphaproteobacteria (C) class.
